# Can Indonesia achieve universal health coverage? Organisational and financing challenges in implementing the national health insurance system

**DOI:** 10.1016/j.ssmhs.2025.100138

**Published:** 2025-12

**Authors:** Dwidjo Susilo, Luh Putu Lila Wulandari, Evi Sukmayeti, Augustine Asante, Stephen Jan, Hasbullah Thabrany, Viroj Tangcharoensathien, Virginia Wiseman, Marco Liverani

**Affiliations:** a Department of Public Policy and Management, Faculty of Social and Political Sciences, Universitas Gadjah Mada, Sleman, Indonesia; b The George Institute for Global Health, University of New South Wales, Sydney, Australia; c Faculty of Medicine and Health, School of Public Health, The University of Sydney, Sydney, Australia; d International Health Policy Programme, Ministry of Public Health, Nonthaburi, Thailand; e School of Public Health & Community Medicine, University of New South Wales, Sydney, Australia; f Kirby Institute, University of New South Wales, Sydney, Australia; g Department of Global Health and Development, London School of Hygiene & Tropical Medicine, London, UK; h Faculty of Medicine, Universitas Udayana, Denpasar, Indonesia; i School of Tropical Medicine and Global Health, Nagasaki University, Nagasaki, Japan; j Faculty of Public Health, Mahidol University, Bangkok, Thailand; k ThinkWell Indonesia, Jakarta, Indonesia; l University of Muhammadiyah Mataram, Mataram, Indonesia

**Keywords:** Indonesia, National health insurance, Universal health coverage, Health financing, Health system reform

## Abstract

Indonesia's National Health Insurance system - the Jaminan Kesehatan Nasional (JKN) - is one of the largest single-payer health insurance schemes in the world, aiming to provide equitable and affordable healthcare to a population of over 280 million. Since its launch in 2014, the JKN has achieved near-universal enrolment, covering 98 % of Indonesians in 2024. However, progress towards universal health coverage – understood as access to the health services people need, when and where they need them, without financial hardship - has been hindered by financing deficits and operational hurdles faced by healthcare providers. In this paper, we examine critical issues affecting the implementation of JKN through the analysis of 20 in-depth interviews and a focus group discussion with government officers and health sector managers at the national and provincial level. Data analysis was guided by a framework combining health systems building blocks and dimensions of access to services. The findings highlight persistent challenges despite the JKN's wide coverage, including difficulties among informal sector workers in paying premiums, regional disparities in service access and health workforce distribution, inefficiencies in provider payment mechanisms, and weak information systems for tracking subsidised members. Interviews also revealed a growing financial and administrative strain on hospitals linked to frequent regulatory changes. To address these issues, we recommend three priority reforms: (1) implement sliding-scale subsidies for informal sector workers; (2) improve provider payment models by introducing cost-sharing for elective services; and (3) adopt participatory policymaking processes to ensure reforms are sustainable and inclusive.

## Introduction

National health insurance systems are critical to the pursuit of Universal Health Coverage (UHC) in low- and middle-income countries (LMICs), as governments aim to reduce financial barriers to healthcare and protect their populations from catastrophic health expenditure (Kutzin, 2013). Yet health care delivery supported through these financing systems, typically funded by general tax revenues or payroll contributions, is often constrained by insufficient budget, fragmented and parallel funding streams, and limited health infrastructure (Cashin and Dossou, 2021, Fusheini et al., 2016, Mishra et al., 2015). Understanding these challenges and their complexities in LMICs is essential for developing policies that promote equitable and sustainable health financing systems.

In 2014, Indonesia launched its National Health Insurance system – the Jaminan Kesehatan Nasional (JKN) - with the goal of achieving UHC by 2019 (MOH, 2014). While the JKN has made significant strides in expanding access to health services (Maulana et al., 2022), many implementation challenges have been documented (Cheng et al., 2025), including a large financing deficit (Prabhakaran et al., 2019), marginal pro-poor distribution of benefits (Asante et al., 2023, Cheng et al., 2022), a high administrative burden on healthcare providers (Banerjee et al., 2021) and variable capacities of local governments in the decentralised public health system (Limasalle et al., 2022). With a population exceeding 280 million, spread across 18,000 islands, national and local governments have struggled to expand their capacity to meet the growing demand for services, especially in disadvantaged and remote areas (Mahendradhata et al., 2017).

Despite a growing body of research on the performance of the JKN, most studies to date have relied on quantitative analyses of administrative data, focusing on financial protection, service utilisation, and equity outcomes. However, there remains limited understanding of the operational realities underlying these outcomes - how policies are implemented on the ground, and how key actors interpret and navigate the system's complex institutional and logistical challenges. This study addresses this gap using a qualitative approach to capture perspectives that are often invisible in quantitative evaluations. By focusing on the lived experiences of those involved in implementing the JKN, it contributes novel insights into the governance, management, and contextual barriers that shape progress towards UHC in Indonesia.

## Overview of Indonesia’s JKN

**Fig. 1:**
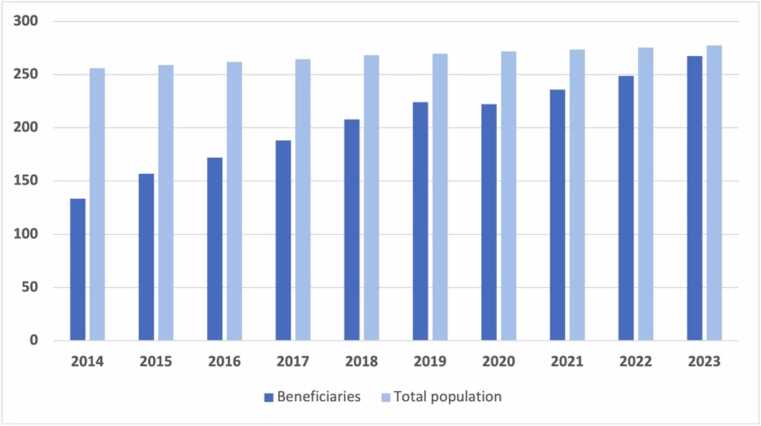
JKN enrolment (2014–2023), in millions. Data sources: Word Bank; National Social Security Council, Indonesia.^1^.

The JKN aims was to provide affordable care to the entire population in Indonesia. At the highest level of policy making, a 2002 amendment to the 1945 Constitution (Article 28 h) stated that “everyone has the right to live in physical and spiritual prosperity, to have a place to live, to have a good and healthy living environment, and the right to obtain health services” (MKRI, 2022). Under this constitutional mandate, the JKN is recognised as one of the most significant equity-driven health reforms undertaken in Indonesia. Nafsiah Mboi, the former Minister of Health (2012–2014) who oversaw the introduction of JKN, said that the main objective of the JKN “is to create a well-integrated, sustainable, accessible, and equitable health system that provides comprehensive, high-quality care to all Indonesians” (Mboi, 2015). Since its launch in 2014, JKN enrolment has increased remarkably (Fig. 1). As of August 2024, it covered more than 276 million people, representing 98 % of the national population (DJSN, 2024).

The JKN is centrally managed by the BPJS Kesehatan, a state-owned agency responsible for the administration of insurance funds (BPJS, 2024). The BPJS Kesehatan is tasked with member registration, collecting contributions, contracting with healthcare providers, and processing claims. It works in collaboration with the Ministry of Health, responsible for setting health policy, regulating services, and ensuring the quality and standards of care provided through the accreditation commissions. The program’s financing is primarily sourced from contributions paid by members and employers, supplemented by government subsidies for low-income households. Community health centres (Pusat Kesehatan Masyarakat, abbreviated as ‘puskesmas’) and other primary care providers are paid in advance through a capitation system (based on the number of registered members), while hospitals are paid on a case-by-case basis according to approved tariffs with the application of the Indonesian Case Base Groups (INA-CBG) for both inpatient and outpatient services, which are set by a technical working group within the Ministry of Health (Chalkley et al., 2022, Mahendradhata et al., 2017).

Since 2018, JKN membership has been mandatory for all Indonesian citizens and legal residents (PR, 2018). Members are categorised into four main groups (Textbox 1), with differing contributions and entitlements to higher or lower classes of hospital accommodation, ranging from VIP private rooms with bathroom, air conditioner and television to shared rooms with fans and minimal facilities. While copayments for covered services are generally not required, members face out-of-pocket (OOP) expenses if they require services beyond standard coverage, such as premium hospital accommodation or uncovered treatments.

Textbox 1 **JKN membership categories. Source: Government of Indonesia.22https://peraturan.bpk.go.id/Details/39833/perpres-no-19-tahun-2016.**

**Table:** 

The JKN classifies its members into distinct categories based on their socioeconomic status. These categories determine how contributions are paid and the level of benefits received: **Contribution Assistance Recipients (Penerima Bantuan Iuran, PBI)**: • Specifically for the poor • Fully subsidized by the government • Enrolment is based on national poverty data • At the end of July 2024, 141.7 million people, accounting for 51.5 % of JKN members, were registered as PBIs **Wage-Earning Workers (Pekerja Penerima Upah, PPU)**: • Includes formal sector workers who receive salaries or wages • Examples: Civil servants, military personnel, police, private sector employees • Their contributions are covered through a salary deduction of 5 %, shared between their employers (4 %) and themselves (1 %) • By July 2024, 55.8 million, or 20.3 % of JKN members belonged to the PPU category **Self-Employed Workers (Pekerja Bukan Penerima Upah, PBPU)**: • Covers self-employed individuals or informal workers • Examples: Small business owners, freelancers • Members are required to pay membership fees, determined by the class of inpatient care chosen • By July 2024, 72.7 million, or 26.4 % of JKN members belonged to the PBPU category **Non-Workers (Bukan Pekerja)**: • Encompasses individuals not formally employed but capable of paying premiums • Examples: Investors, retirees, veterans and other non-working individuals • By July 2024, 5.1 million, or 1.8 % of JKN members belonged to the Bukan Pekerja category

The JKN provides a comprehensive package of health services that covers both primary and secondary healthcare, including maternity services, surgical procedures, diagnostic services, and medicines. Within fourteen days of enrolment, JKN members are required to register with a primary health care provider of their choice, including puskesmas and other first-level government facilities, private clinics, and general practitioners in the public and private sector. If more specialised care is needed, the primary care provider will refer the patient to a hospital in class D (basic medical services), C or B (secondary referral hospitals), from where the patient can then be referred to a top tertiary hospital (class A), if necessary.

## Methods

### Study design

This study is part of a larger evaluation of equity in health systems financing in Indonesia (the ENHANCE project) using quantitative techniques including financing incidence or progressivity analysis and benefit incidence analysis (Wiseman et al., 2018). Here, we employed a qualitative research design to explore the implementation of the JKN from the perspective of key stakeholders. The qualitative approach was chosen to capture insights into good practices, challenges, and contextual factors that influence the operation of the JKN. The study design was guided by the six WHO health system ‘building blocks’ (World Health Organization (WHO), 2010a, World Health Organization (WHO), 2010b) - service delivery, health workforce, health information systems, access to essential medicines, financing, and governance – together with key dimensions of access to care, namely geographic accessibility, affordability, and availability (Oliver and Mossialos, 2004). These complementary frameworks provide a systematic, well-established approach for assessing progress towards UHC, allowing us to examine both the organisational, service delivery and financing challenges and the achievements of Indonesia’s JKN.

### Study setting and participants

Semi-structured interviews were used as the primary data collection method, allowing for flexibility in the discussion while ensuring that core topics related to JKN implementation were covered. Interviews were conducted in the capital Jakarta and three provinces (Central Java, East Kalimantan, and Lampung). Jakarta was included to represent the national level, as it hosts key policy institutions and national-level stakeholders. The selection of these provinces was intended to capture variations in healthcare access, provider capacity, and service delivery that may influence functioning of the JKN. Central Java was chosen as a populous province with relatively well-developed health infrastructure; East Kalimantan as a resource-rich but geographically dispersed province; and Lampung as a predominantly rural province with lower health service capacity. Participants were purposively selected to include a wide range of stakeholders involved in policy development and implementation. These included government officers, BPJS managers and health sector managers at the central and provincial level, representatives of professional associations, and hospital directors. Participants were identified using the professional network of the researchers involved in this study and snowball sampling. Inclusion criteria were: (1) direct involvement in JKN policymaking, management, or service delivery; (2) holding a position in government, the BPJS, a professional association or a health facility with decision-making or operational responsibility related to JKN; and (3) willingness to participate. The interviews were conducted in 2020, followed by a focus group discussion in August 2023 with different participants to verify issues that emerged during the interviews and provide updated information on recent developments. The FGD involved stakeholders from health organizations, government bodies, advocacy groups, healthcare providers, business associations, labour unions, and the pharmaceutical industry.

### Data collection

The interviews with stakeholders focused on the following topic areas: (1) experiences with implementing the JKN, including perceived successes and challenges to health service delivery, access to services by members; (2) the financial and administrative burden of the JKN on healthcare providers; (3) suggestions for improving the implementation approach. Depending on the expertise, responsibility and experience of each informant, the interview content was adapted to capture different health system blocks and access dimensions following the conceptual framework described above. The interviews and the FGD were conducted in Bahasa Indonesia by DS and two researchers, audio recorded, transcribed, and then translated into English; extensive notes were also taken during and after the interviews. They lasted one hour on average - ranging from 30 to 90 min - and were conducted either in person or via phone/video call depending on participants’ availability. The FGD was conducted in person in two sessions (morning and afternoon) on 16th August 2023, in Jakarta. Documentary materials and publications such as policy papers and reports were reviewed at various stages to provide context, deepen understanding of the research themes, and clarify specific points.

### Data analysis

The analysis involved familiarization with the data, coding, searching for themes, reviewing themes, and defining and naming themes (Maxwell, 2012). Interview transcripts and notes were read multiple times to gain an overall understanding of the content. After familiarization, the data were systematically coded using both inductive and deductive approaches. The initial analytical framework was based on the health system building blocks and access domains outlined above. This framework was applied for selective coding of the dataset, facilitated by NVivo 13 software (QSR International, 2020). Open coding was also employed to allow for a broader examination of the data and to uncover emerging themes. A team of researchers independently coded the transcripts to ensure reliability, and any discrepancies in coding were discussed to clarify interpretation.

### Ethical considerations

This study received ethical approval from the Commission of Research Ethics and Community Health Service, Faculty of Public Health, University of Indonesia (Ke-t 602/UN2.F10/PPM.00.02/2019) and the London School of Hygiene and Tropical Medicine (13773). Written informed consent was obtained from all participants prior to the interviews and the FGD, and participants were assured of confidentiality and the voluntary nature of their participation. All identifying information was anonymized during transcription and data storage, and interviews were stored securely to protect participant privacy.

## Results

A total of 20 participants were interviewed, with different roles and responsibilities in planning or implementation of the JKN, as summarised in Table 1. The FGD was joined by 17 stakeholders in Jakarta, including from the BPJS, Ministry of Health and other government bodies (i.e the Presidential Office, the National Social Security Council, the National Poverty Alleviation Team), professional associations of healthcare providers, trade unions, and business organisations. In the sections below, we present the main findings, organised around the health system building blocks and access domains described above. Key points from the interviews and anonymised citations are referenced by the unique identifiers included in Table1.

**Table 1:** Interview participants by location, type of organization and identifier.

**Institution**	**ID**
**Jakarta**	
BPJS	JKK-BPJS01, JKK-BPJS J02, JKK-BPJS03
Central government	JKK-GOV01, JKK-GOV02, JKK-GOV03
Indonesian Medical Association	JKK-IMA
Indonesian Hospital Association	JKK-IHA
Indonesian Pharmacist Association	JKK-IPA
**Central Java (Semarang & Metro)**	
Provincial Health Office	CJ-MOH01
Municipal Health Office	CJ-MOH02
Government hospital	CJ-HH01
Private hospital	CJ-HH01, CJ-HH03
**East Kalimantan**	
Provincial Health Office	EK-MOH
Private hospital	EK-HH
**Lampung**	
Municipal Health Office	L-MOH
Government hospital	L-HH01
Private hospital	L-HH02, L-HH03

### Access to services

Many interview participants acknowledged that the implementation of the JKN had positive impacts on strengthening health service delivery. In all three provinces, new health facilities were being developed or upgraded to accommodate larger numbers of insured patients. In East Kalimantan, local authorities had received support from the central government to build new puskesmas, while other puskesmas were upgraded to the status of class D hospitals (EK-MOH). In Lampung, two public hospitals had been renovated, providing additional services covered by the JKN such as speech therapy and geriatric services (L-MOH).

However, the representative of a hospital association complained that inpatient beds especially in ordinary wards was inadequate due inefficiencies in the information system for ward management and priority given to patients with premium accommodation or private insurance, resulting in long waiting times for other JKN members:

“What we often advocate is more availability of inpatient beds. When a patient needs to be hospitalized, beds are full.” (JKK-IHA I)

#### Geographic accessibility

Participants emphasized that geographic accessibility of health facilities is central to achieving UHC in Indonesia. Since the launch of the JKN, the network of health facilities has been expanded to increase coverage and, in some areas, the road infrastructure to reach them has improved. In Lampung, for example, it was reported that one hospital financed the construction of a bridge, reducing the travel time from the surrounding communities from 3 hours to 30 min (L-HH03). In other locations, the accessibility of health facilities and associated costs remained a significant challenge:

“They have the BPJS card from the government, but their houses are 3 or 4 hours away from here. When they come to this clinic, they must rent a car and pay 600 thousand rupiah.” (L-HH0)

“The communities complain about the location of referral hospitals. For some people, hospitals are too far from their place of residence” (CJ-MOH02)

The director of a haemodialysis clinic in Lampung further commented that “the dialysis is free, but the costs of transportation, accommodation, eating and drinking are high” (L-HH02), especially for those who live in remote areas. He then explained that these costs are higher for some categories of chronic patients such as end-stage renal patients, who require two or three dialysis sessions per week for end-stage renal treatment:

“Haemodialysis patients could incur travel costs of more than 1 million rupiah a week, if they must travel to the hospitals two times a week to receive treatment” (L-HH02).

#### Affordability of JKN premiums for PBPU members

Informants acknowledged the success of the JKN in terms of expanded coverage. However, concerns emerged about the continued enrolment of PBPU participants. As described above, the PBPU category includes self-employed individuals and informal sector workers who do not qualify as poor but do not receive regular wages from an employer, so they are required to pay for JKN membership. In Lampung, the director of a private clinic stressed that many PBPU members have unstable and unpredictable income, making it difficult for them to consistently pay their monthly premiums, resulting in suspension of JKN membership and access to eligible services. If a PBPU member fails to pay their contribution, the system sends a warning to providers. During the first three months of non-payment, members can still access outpatient care but must pay a penalty. If payments remain overdue for more than three months, they lose all coverage, including both outpatient and inpatient services.

“For those who do not have enough money, paying BPJS contribution is challenging. And they need to pay for all family members listed in the family card. Many people cannot afford this” (L-HH03)

The requirement for PBPU participants to repay any outstanding arrears to reactivate and maintain membership was identified as a major factor contributing to high dropout rates in the PBPU category (L-HH02, L-MOH). During the FGD, a health officer shared the story of a couple who were unable to regain membership due to their inability to pay BPJS arrears during the COVID-19 pandemic.

“During the pandemic, they had no income because their business was down. The household savings were used to survive. After the pandemic, their business improved and they wanted to pay JKN contributions. Unfortunately, they had to pay a debt of around 11 million rupiah as a requirement for reactivating their membership. Negotiations have been made in order to reduce their debt, but they were only given the opportunity to pay in installments, without any debt relief. So, they decided not to continue participating in JKN because they could not afford to pay for the arrears” (FGD).

According to the insurance policy, PBPU members are eligible to register as PBI members if their income status worsens. However, access to PBI is granted only after settlement of any outstanding payments with the PBPU (Government of Indonesia, 2013). Informants explained that the requirement to clear arrears (either for continuation of PBPU status or transfer to PBI membership) places an additional financial burden on individuals who are already struggling, preventing the most vulnerable from accessing the health services they need.

“Some residents of Semarang City are registered under the PBPU scheme, but they are poor. Many of them are in arrears. And we cannot enrol them in the PBI scheme because of the outstanding arrears” (CJ-MOH02)

“We want to support them through the government subsidy, but this is difficult because the arrears must be paid first. They don’t have much money and we cannot pay for the arrears either” (L-MOH)

“We want to eliminate the arrears repayment, but we cannot do this because debt relief is not allowed under current regulation” (FGD)

### Health workforce

Constraints to health service delivery were also discussed in relation to the inequitable distribution of the health workforce, especially in remote rural areas – another key barrier to achieving UHC in Indonesia. In urban centres too, hospital managers reported challenges to the employment and retention of specialised doctors:

“This is a provincial hospital, but we are still lacking health professionals, particularly specialists” (L-HH01)

“The biggest challenge in my opinion is how we provide health services in health facilities with inadequate number of health workers” (JKK-BPJS03)

As a hospital director in Semarang explained, insufficient availability of specialised doctors resulted in long waiting times for JKN patients undergoing cancer treatment and other specialist care. When discussing these challenges, the representative of a professional organization stressed that different salary levels across hospital types affected the distribution and availability of doctors in the lower tier. As he explained, skilled doctors are drawn to type A or B hospitals that offer higher compensation by the BPJS, creating an imbalance where hospitals C and D struggle to attract and retain qualified personnel (JKK-IHA):

“In my view, differences in compensation constitute structural discrimination because the procedures are performed by doctors with the same competency and certification, using the same standard equipment and medications. The only difference is the classification of the hospital type” (CJ-HH03)

### Access to medicines

The supply of medicines and medical equipment under the JKN is provided through an online ‘e-Catalog’, a centralised procurement system managed by the government to streamline the purchasing process. Pharmaceutical companies and suppliers register their products in the e-Catalog in accordance with the quality standards set by the Ministry of Health. Public hospitals and other health providers can find and purchase listed products through online procurement. This system is also available to the private sector, although it was explained that private providers prefer to buy directly from trusted wholesalers (JKK-IPA).

The e-Catolog is in principle an effective strategy for promoting transparency in pricing, quality assurance, and maintaining cost efficiency. However, barriers to accessing medicines were reported in remote areas due logistical challenges and the high costs of shipment and delivery.

“The shortage of drugs in several areas of East Kalimantan is not due to lack of budget, but because of challenging logistical arrangements. The suppliers are unable to deliver the drugs to remote locations” (EK-MOH)

“The cost [of shipment] is higher than the price of the medicine, especially in areas with poor infrastructure” (JKK-IPA)

Another limitation discussed during the interviews is that expensive medicines such as chemotherapy treatments cannot be purchased at standardised prices through the online system. The JKN program reimburses hospitals at fixed rates for these medications, often lower than the market price or the cost at which hospitals procure them (JKK-IPA). As a result, many hospitals struggle to recover the costs of purchasing these drugs which can discourage them from stocking essential medications, potentially limiting patient access to necessary treatments:

“We are trying to be as efficient as possible by using drugs that comply with the national formulary. If they don’t, we will lose money”. (CJ-HH01)

### Information systems

Registering JKN members has been a formidable data management challenge for the BPJS. In the interviews, participants emphasized specific challenges associated with identifying and registering the low-income groups to PBI status. The identification details of poor households are recorded by the Statistical Bureau of the Ministry of Social Affairs, based on information inputs from local offices. However, two informants mentioned that many poor households are not included in the poverty database, while others are registered but unaware of their JKN rights and therefore do not use the services covered by the scheme. It was also noted that undocumented migrants cannot be registered as residents of the place of migration (L-HH02); therefore, they are not eligible for JKN services.

“Not everyone has an identification card. If they have no ID, they cannot register to JKN” (Jkk-IHA)

“Many poor people don’t know they are registered as PBI, so they don’t get the PBI card for JKN membership” (FGD).

Conscious of these constraints, some local governments have stepped in to support those who slip through the administrative net. In Semarang, for example, the local government provided matching funds to pay the premium for residents and migrants who were not covered by JKN, regardless of their socio-economic status (CJ-MOH02). Similarly, the provincial government in Lampung and Central Java contributed some funds to the JKN, with allocation depending on the fiscal capacity of each administration (L-MOH):

“The city of Semarang does not discriminate between poor and rich, and the city government provides PBI by registering in class C. But subsidies depend on the fiscal capacity of each local government.” (JKK-IHA)

The registration of newborn babies emerged as another important information challenge. As per the Presidential Regulation 82 (2018), newborn babies of PBPU members must be registered with the BPJS Kesehatan within 28 days after birth, while PBI members must register their newborn with the local Population and Civil Registration Office (Disdukcapil) within 60 days. However, informants explained that frequent and long delays in registration affected access to health services and threatened financial protection of families.

“Midwives did not know at all that these policies or regulations exist. Those who gave birth in the hospitals were on average not PBI members, not the poor. According to the regulations, children born to mothers who are PBI members automatically become PBI participants. However, from the data, it turns out that there are still many babies born by PBI members who have not been automatically recorded in the system” (FGD)

### Financing and provider payments

After the first five years of implementation, the JKN incurred a cumulative deficit of IDR 50 trillion (US$ 3.5 billion), leading to approval by the World Bank in 2021 of a US$400 million Program-for-Results loan and a US$2.33 million grant (World Bank, 2021). This large deficit has imposed tight budget management and efficient cost control by the BPJS, resulting in financial pressures on health facilities, even if they receive additional funding from the government to sustain operations and international donors for specific programmes. One informant in the Ministry of Health explained:

“Now we know that the initial package of services offered by the JKN was not sustainable (financially), leading to a reduction in services… Because of the deficit, healthcare providers are squeezed and must become more efficient. Ultimately, BPJS transfers the financial risk to service providers.” (CJ-MOH02)

In East Kalimantan the director of a private clinic with about 1000 JKN members said that the amount of capitation received - IDR 8000 (US$ 0.50) per participant, paid each month (equivalent to US$ 6 per person per annum) - was insufficient to recover the cost of doctors and medicines.

“Actually, the eight thousand rupiah (monthly) capitation is rather minimal because it includes doctor fees and drugs. It has almost nothing to do with it. We are fortunate that this building is owned by the clinic, so we don’t have to pay for the rent“ (EK-HH)

Similar pressures affected hospitals also due to discrepancies between the INA-CBGs tariffs paid by the BPJS for inpatient services and the actual costs incurred by hospitals. Delays in reimbursement and audits resulted in disruptions to hospital cash flows.

“In addition to tariff issue, several other things that are also quite disruptive to hospital financing are pending claims, different administrative procedures across BPJS branches, and retrospective claim reviews.” (JKK-IPA).

“… the private type B hospitals have the same complaint, that the INA CBGs tariff is not sufficient to recover their costs.” (CJ-HH01)

### Governance

Since the introduction of the JKN in 2014, the management and financing of this large insurance scheme have posed policy and implementation challenges, requiring many adjustments to budgeting, procurement processes, and information systems. In the interviews, hospital managers and health officers explained that frequent amendments of these regulatory provisions and practices had implications for financial planning and the administration of health facilities, leading to inefficiencies and disruptions to service delivery.

“Changes in regulations that are so frequent and retroactive that are detrimental to hospitals” (CJ-HH01)

“BPJS regulations change very often, and sometimes, we are informed only when the new rules are already in effect” (CJ-MOH02)

It was also noted that the current regulation of referral practices, where puskesmas can only refer patients to type C or D hospitals, resulted in inequities. While this policy led to a substantial increase in patient loads in type C and D hospitals, informants explained that the number of patients visiting type A and B hospitals decreased, encouraging hospital managers in type B hospital to downgrade their classification to receive more referrals:

“… there are some hospitals like that want to downgrade to C, especially the private sectors because they want to catch patients referred from primary care facilities.” (L-HH02)

## Discussion

This study explored the perspectives and experiences of different categories of stakeholders regarding the implementation of the JKN in Indonesia. The findings reveal both the significant progress made in expanding coverage and access to healthcare services and the ongoing challenges, highlighting critical issues that need further consideration for research and policy. Addressing these gaps - particularly in service quality, equity, and financial protection - will be essential if Indonesia is to realise UHC through the expansion of the JKN scheme.

First, the expansion and upgrading of health facilities reported in the three provinces demonstrate the government’s commitment to accommodating the growing number of and demands for services by JKN participants. The construction of new facilities and renovations of existing hospitals and puskesmas, as seen in East Kalimantan and Lampung, are critical steps in strengthening the local health infrastructure and ensuring that more people have access to the care they need. However, stakeholders highlighted persisting disparities in the availability of healthcare providers and essential resources, such as primary care, hospital beds and specialised services. These constraints are particularly severe in remote and rural areas, where access to health facilities, medicines, and quality services is often hindered by distance, high transportation costs, and logistic challenges - with the greatest impact on patients requiring chronic care such as haemodialysis services. Similar issues have been observed in other LMICs, where efforts to achieve universal health coverage are not supported by corresponding investments in human resources and health service delivery in hard-to-reach areas (Ahmed et al., 2024, Reich et al., 2016). In other countries, such as Thailand, the full geographical coverage of primary care services nationwide (including district hospital and sub-district health centres) has been the foundation of UHC, resulting in a great reduction in child mortality gaps and other key indicators across rich and poor provinces (Tangcharoensathien et al., 2018; Gruber et al. 2014). Higher compensation for physicians in type A and B hospitals should be reconsidered as doctors will leave lower-level hospital and primary care and widens inequity gap. Financial incentives should instead be linked with hardship or workload intensity.

Second, despite a remarkable increase in the number of participants (Fig. 1), our study indicates that some poor households are still unable to access subsidised services either because they are not included in the registration system or remain unaware of their eligibility. In keeping with this finding, a recent survey in Indonesia found that enrolment in JKN - as reported by participants - decreased in lower socio-economic groups, with the authors hypothesising that “the government is paying premiums for some poorer people without their awareness” (Pratiwi et al. 2021). These gaps undermine the program’s goal of equitable coverage and emphasize the need for improved information systems and more inclusive policies, as found in other countries. For example, in Burkina Faso, similar challenges in identifying poor households for enrolment in subsidised community health insurance have been documented (Savadogo et al. 2015).

Third, informal workers in the PBPU category – the so called ‘missing middle’ (Augustina et al., 2019) – struggle to maintain regular JKN membership due to unstable incomes and inability to pay the monthly premium, while the requirement to settle arrears to reactivate their membership, or transfer to PBI status if eligible, was seen as a key determinant of high dropout rates. In fact, at the end of July 2024, as many as 51 million members were registered as inactive participants due to payment arrears (DJSN, 2024). This finding reflects similar challenges in other LMICs, such as India, where those who are engaged in informal work are often not able to afford regular premium payments but are not poor enough to benefit from state subsidized contributions to insurance premiums (Mahal et al. 2024). Our study further indicates that the inability to transfer those with outstanding arrears to the government-subsidized PBI scheme exacerbates vulnerability, leaving many without sufficient financial protection. Reforms that provide more flexible payment schedules are needed to ensure continuous coverage for these individuals. In the future, a deeper or more significant reform could be considered - specifically, merging the PBPU with the PBI group, so that informal workers are also covered by public subsidies. when government’s fiscal space allows. The administrative cost of monthly premium collection for millions of informal workers is another reason to consider further policy change.

Fourth, the large deficits reported indicate that the current financing sources are insufficient to cover the costs of services. As an upper-middle-income country with a GNI per capita of US$ 4870 as of 2023, a bold policy decision was made in 2013 to finance subsides for the poor and near-poor from general taxation. However, this approach requires strong governance mechanisms and expanded fiscal capacity. Fiscal space can be improved through tax reforms, as tax revenue currently accounts for 10.4 % of GDP - a figure that remains well below the Asia and Pacific average of 19.3 % and the OECD average of 34.0 % (OECD, 2024). Furthermore, delays in reimbursement to hospitals and insufficient capitation rates in primary care highlight the need for adjustments in financing and budget management, ensuring that health facilities are adequately compensated for the services they provided, particularly in case of expensive treatments. In this context of financing gaps, the ‘missing middle’ should be given particular attention. When benefits of PBPU members are suspended or terminated, they face increased health and economic risks and no longer help to sustain the national health sector. To address this issue, the requirement for full payment of arrears before membership reactivation could be reconsidered, as it may undermine efforts to maintain comprehensive coverage and financial with their contributions protection. At the same time, incentives to increase the participation of PBPU members could be devised, such as ‘sliding-scale’ subsidies where the amount of subsidy provided decreases as the recipient's income or ability to pay increases (Shewamene et al. 2021).

Experiences from other countries demonstrate that alternative health financing and subsidisation models can help sustain coverage among informal sector workers. Thailand’s approach to achieving UHC offers valuable insights. Prior to 2002, informal sector workers were covered under a publicly subsidized health insurance scheme, in which households contributed 50 % of the premium while the government subsidized the remaining 50 %. However, in 2002, this scheme was replaced by the Universal Coverage Scheme (UCS), a tax-financed insurance system providing access to healthcare to all citizens who were not covered under the Civil Servant Medical Benefit Scheme (CSMBS) or the Social Health Insurance (SHI) scheme for employees in the private sector (Tangcharoensathien et al. 2018). The UCS provides seamless coverage transitions to ensure continuity of care: SHI members who become unemployed or retire are automatically transferred to the UCS, while dependents of CSMBS lose eligibility at age 20 but are enrolled in the UCS unless they qualify for other membership. On the other hand, individuals who gain employment in the private or public sector transition from the UCS to the SHI or CSMBS membership, respectively. Remarkably, Thailand’s achievement of UHC - at a time when its gross national income per capita was only USD 1980 - demonstrates that a tax-financed, non-contributory model can be both financially feasible and administratively effective in a middle-income setting. For Indonesia, where the informal sector remains large and contribution collection is costly and inconsistent, Thailand’s model underscores the potential of adopting a non-contributory approach to close coverage gaps and reduce fragmentation (Tangcharoensathien et al. 2018).

Lastly, future JKN reform should be informed by participatory consultations with all relevant stakeholders - including public and private healthcare providers, local governments and the communities - and guided by updated evidence about the cost of services and their utilization rates. As described, frequent changes in BPJS regulations, often implemented retroactively, have led to confusions and inefficiencies in service delivery. Hospital managers and health officers highlighted the administrative burden imposed by these frequent regulatory shifts, which disrupts their financial and operational routines. Therefore, healthcare providers will need sufficient time to adapt to regulatory change, along with clear and timely communication from BPJS. A more consultative approach to policy development will also ensure that diverse perspectives are considered, leading to policies that are more inclusive, accepted, and aligned with local needs and capacities.

## Conclusion

The implementation of JKN has expanded access to healthcare across the study provinces; however, key challenges remain that threaten the programme’s equity and financial sustainability. These include persistent geographic disparities in service availability, premium affordability barriers for informal sector workers, shortages of specialised services, and administrative inefficiencies. Addressing these issues is essential to realising the central goal of JKN: achieving universal health coverage for all Indonesians.

To this end, in the discussion we have identified key priority areas for further policy development, summarised in Textbox 2. While these recommendations are informed by rich qualitative insights, a limitation of this study is the timing of data collection, with interviews conducted in 2020 and focus group discussions in 2023. Although the findings remain relevant to understanding persistent implementation challenges, they do not capture the effects of more recent policy and regulatory changes to the JKN scheme. These include reforms aimed at improving financial sustainability - such as increased government subsidies and earmarked cigarette tax revenues - as well as the growing digitalisation of JKN service delivery through the Mobile JKN app, which now offers online registration, premium payments, and telemedicine services. Future research will be needed to evaluate how these developments affect coverage, service delivery, and equity. Another limitation is that the study was conducted in three provinces so the results may not reflect variations in socioeconomic conditions, health service delivery, or policy implementation nationwide.

Textbox 2 **Policy recommendations.**

**Table:** 

1. Enhance financial protection for informal sector workers through a stepwise targeted approach: begin with sliding-scale subsidies and flexible payment schedules, and ultimately merge PBPU with the PBI scheme where fiscal space allows, to ensure continuous coverage, reduce dropouts, and strengthen financial risk protection. 2. Strengthen health service delivery in underserved areas by expanding and upgrading facilities, training and redistributing the health workforce (including specialists), and aligning financial incentives with hardship postings and workload. 3. Improve coverage of poor and near-poor households by strengthening identification systems, leveraging digital technologies, and enhancing outreach to ensure all eligible households are enrolled in subsidised schemes and informed of their entitlements. 4. Secure sustainable financing by expanding fiscal space through tax reforms, earmarked revenues, and efficiency measures (reducing low-value care), and by ensuring timely reimbursement and adequate provider payment rates, especially in primary care. 5. Streamline regulatory processes and information systems by reducing administrative burdens on providers, improving communication of regulatory changes, and strengthening oversight of subsidised population coverage and performance. 6. Adopt a participatory approach to policy development by engaging healthcare providers, local governments, civil society, and communities in reform design and implementation to ensure feasibility, inclusiveness, and responsiveness to local needs.

## CRediT authorship contribution statement

**Marco Liverani:** Writing – original draft, Supervision, Formal analysis. **Evi Sukmayeti:** Formal analysis. **Viroj Tangcharoensathien:** Writing – review & editing. **Virginia Wiseman:** Writing – review & editing, Supervision, Resources, Funding acquisition, Conceptualization. **Dwidjo Susilo:** Writing – review & editing, Investigation, Formal analysis, Data curation. **Stephen Jan:** Writing – review & editing. **Hasbullah Thabrany:** Writing – review & editing, Resources, Conceptualization. **Luh Putu Lila Wulandari:** Formal analysis, Data curation. **Augustine Asante:** Writing – review & editing.

## Funding

This study was supported by a grant from the Health Systems Research Initiative in the UK, jointly funded by the Department for International Development (now Foreign and Commonwealth Development Office), the Economic and Social Research Council, the Medical Research Council and the Wellcome Trust (MR/P013996/1).The funders were not involved in study design, in the collection, analysis and interpretation of data, in the writing of the articles; or in the decision to submit for publication.

## Declaration of Competing Interest

The authors declare that they have no known competing financial interests or personal relationships that could have appeared to influence the work reported in this paper.

## Footnotes

1 Dewan Jaminan Sosial Nasional (DJSN); djsn.go.id
